# Clinical and Hemodynamic Effects of Pulmonary Artery Denervation in Pulmonary Hypertension Despite Optimized Pharmacotherapy: An Updated Systematic Review and Meta-Analysis

**DOI:** 10.3390/jcm15072619

**Published:** 2026-03-30

**Authors:** Elif Ijlal Cekirdekci, Lutfi Cagatay Onar

**Affiliations:** 1 Department of Cardiology, University of Kyrenia, Kyrenia 99320, Cyprus; 2 Department of Cardiovascular Surgery, Republic of Turkey Ministry of Health, Dr. Ismail Fehmi Cumalioglu City Hospital, Tekirdag 59020, Turkey

**Keywords:** functional outcomes, hemodynamics, meta-analysis, pulmonary artery denervation, pulmonary hypertension, right ventricular function

## Abstract

**Background**: Pulmonary hypertension (PH) remains a progressive and life-threatening condition despite advances in targeted pharmacotherapy. Pulmonary artery denervation (PADN) has emerged as a novel interventional strategy aimed at modulating sympathetic overactivity and improving pulmonary vascular hemodynamics. **Methods**: A comprehensive search of PubMed, EMBASE, Scopus, Web of Science, and the Cochrane Library was conducted through December 2024. Randomized clinical trials and prospective observational studies assessing PADN in PH were included. Primary endpoints were changes in outcomes from six-minute walk distance (6MWD), mean pulmonary artery pressure (mPAP), pulmonary vascular resistance (PVR), cardiac output (CO), and right ventricular function parameters. Secondary outcomes included clinical worsening, rehospitalization, transplantation, and all-cause mortality. Random-effects models were used to calculate pooled mean differences (MDs) and odds ratios (ORs) with 95% confidence intervals (CIs). Subgroup analyses were performed according to pulmonary hypertension phenotype and study design, and sensitivity analyses were conducted to assess robustness of pooled estimates. **Results**: Nine studies involving 454 patients were included. PADN significantly improved functional capacity (6MWD: MD = 92.03 m; 95% CI 46.37–137.68; *p* < 0.001) and reduced mPAP (MD = −11.84 mmHg; *p* < 0.001) and PVR (MD = −4.88; *p* < 0.001). Cardiac output increased significantly (MD = 0.55 L/min; *p* < 0.001), with improvements observed in right ventricular functional indices. PADN was associated with a lower risk of clinical worsening (OR = 0.30; *p* = 0.001) and rehospitalization (OR = 0.07; *p* < 0.001), whereas no significant difference was observed in all-cause mortality (OR = 0.53; *p* = 0.12). Considerable heterogeneity was observed across functional and hemodynamic outcomes, reflecting variability in study design, patient populations, and PADN techniques. **Conclusions**: PADN significantly improves exercise capacity and pulmonary hemodynamics in patients with PH, particularly in those with persistent symptoms despite medical therapy. Although PADN reduces clinical deterioration and rehospitalization, its impact on long-term survival remains uncertain. Further large-scale, multicenter randomized trials are needed to better define optimal patient selection and determine long-term clinical benefit.

## 1. Introduction

Pulmonary hypertension (PH) is a progressive and multifactorial cardiopulmonary disorder characterized by sustained elevation of pulmonary arterial pressure and pulmonary vascular resistance, ultimately leading to right ventricular (RV) failure and premature mortality [1]. According to the 2022 guidelines of the European Society of Cardiology and the European Respiratory Society, PH is defined as a mean pulmonary artery pressure (mPAP) ≥ 20 mmHg measured by right heart catheterization [1,2]. Beyond its hemodynamic definition, PH represents a complex pathobiological spectrum involving endothelial dysfunction, inflammation, vascular remodeling, and maladaptive RV–pulmonary arterial coupling [3]. Despite advances in diagnosis and management, PH continues to be associated with significant morbidity and impaired functional capacity across age groups and clinical subtypes [4]. PH is associated with high hospitalization rates, reduced quality of life, and substantial healthcare utilization despite modern combination therapies [1,2].

Current treatment strategies primarily target the endothelin, nitric oxide, and prostacyclin signaling pathways. Although these therapies improve symptoms and delay disease progression, a substantial proportion of patients remain symptomatic or experience clinical worsening despite optimized combination therapy. A therapeutic ceiling effect is frequently observed, with a substantial subgroup of patients classified as non-responders despite escalation to combination and triple therapy. Consequently, there is increasing interest in therapeutic strategies that address additional mechanistic contributors beyond vasodilation alone. Pulmonary artery denervation (PADN) has emerged as a novel interventional approach aimed at modulating autonomic nervous system activity within the pulmonary arterial wall [5,6,7].

Experimental studies have provided mechanistic support for this strategy. PADN targets the sympathetic afferent and efferent nerve fibers located within the adventitial layer of the pulmonary artery, which are involved in neurohumoral regulation of pulmonary vascular tone and remodeling [5,6,7,8,9,10,11,12]. In animal models of pulmonary hypertension, PADN has been shown to reduce pulmonary artery pressure and attenuate vascular remodeling [8,11]. Moreover, autonomic modulation appears to influence right ventricular function and local renin–angiotensin–aldosterone system activity, suggesting a broader neurohumoral impact beyond isolated hemodynamic changes [9,12]. Collectively, these findings underscore the biological rationale for modulating sympathetic overactivity as an adjunctive therapeutic target in pulmonary hypertension.

Translational application of PADN into clinical practice began with the first-in-man PADN-1 study, which demonstrated reductions in mPAP and pulmonary vascular resistance (PVR) together with improvements in exercise capacity [13]. Subsequent phase II data confirmed sustained hemodynamic and functional benefits in selected patient populations [14], and further analyses highlighted the potential impact of PADN on right ventricular mechanics [15]. Building upon these early findings, several randomized and prospective trials have evaluated PADN across different PH phenotypes, including pulmonary arterial hypertension and PH associated with left heart disease [16,17,18,19,20]. Additionally, PADN has been investigated in patients with residual pulmonary hypertension following pulmonary endarterectomy [21,22,23]. While many of these studies reported improvements in six-minute walk distance and pulmonary hemodynamics, variability in study design, patient selection, and follow-up duration has limited definitive conclusions.

A recent meta-analysis suggested potential benefits of PADN in improving functional and hemodynamic parameters; however, the analysis was limited by the evolving evidence base and the availability of contemporary randomized data [24]. However, existing meta-analyses are limited by small sample sizes, lack of contemporary sham-controlled randomized trials, heterogeneous PADN techniques, and insufficient evaluation of long-term clinical outcomes [24]. With the increasing availability of contemporary trials and longer-term outcome data, a comprehensive and updated synthesis of the evidence is needed.

Therefore, we conducted a comprehensive systematic review and meta-analysis of randomized and prospective observational studies to evaluate the effects of PADN on functional capacity, pulmonary hemodynamics, right ventricular function, and clinical outcomes in patients with pulmonary hypertension refractory to medical therapy, with the aim of clarifying its therapeutic role, patient selection, and potential long-term clinical impact.

## 2. Methods

### 2.1. Search Strategy

A systematic literature search was conducted in PubMed, EMBASE, Scopus, Web of Science, and the Cochrane Library to identify relevant studies published through 24 December 2024 [25,26]. Search terms included combinations of “pulmonary arterial hypertension”, “pulmonary hypertension”, “arterial hypertension, pulmonary”, “hypertension, pulmonary arterial”, and “pulmonary artery denervation” [27]. Reference lists of retrieved articles, reviews, and editorials were manually screened to identify additional eligible studies. Only English-language publications were included due to feasibility considerations. Duplicate records were removed using Paperpile and Rayyan, and the remaining studies were independently screened for eligibility based on titles and abstracts by two reviewers (EIC and LCO) in accordance with established methodological standards [28]. As Supplementary Materials you can find the PRISMA checklist [26].

### 2.2. Eligibility Criteria

Studies were eligible for inclusion if they:

- Reported on adult patients with pulmonary hypertension who underwent pulmonary artery denervation (PADN) [13,14,15,16,17,18,19,20,21,22,23].
- Were randomized controlled trials (RCTs) or prospective observational studies [16,17,18,19,20,21,22,23].
- Reported outcomes related to functional capacity, pulmonary hemodynamics, right ventricular function, or clinical outcomes [8,9,10,11,12,24]. Refractory disease was defined as persistent WHO functional class III–IV symptoms despite optimized guideline-directed medical therapy, including combination therapy where applicable.

Exclusion criteria included:

- Animal studies or preclinical research [8,11].
- Case reports, case series without prospective follow-up, or conference abstracts without full text [24].
- Duplicate publications or studies lacking sufficient data on outcomes of interest [25,26].

### 2.3. Data Extraction

Two reviewers (EIC and LCO) independently extracted the following data using a standardized form:

- Study characteristics: Year of publication, design, enrollment period, and follow-up duration [16,17,18,19,20,21,22,23].
- Patient characteristics: Age, sex distribution, and WHO PH classification [1,2].
- Intervention details: Type and technique of PADN [5,6,7,13,14,15].
- Outcomes: Primary endpoints (6MWD, mPAP, PVR, CO, TAPSE, RV FAC, and NT-proBNP) [8,9,10,11,12], and secondary endpoints (clinical worsening, rehospitalization, lung transplantation, and all-cause mortality) [24].

Discrepancies between reviewers were resolved by discussion or consultation with a third reviewer. Inter-reviewer agreement was assessed using Cohen’s kappa coefficient, with discrepancies resolved by consensus or third-reviewer adjudication.

### 2.4. Quality Assessment

The ROBINS-I tool was used to evaluate the risk of bias in non-randomized studies, while the Cochrane Risk of Bias tool was applied for RCTs [29]. Each study was independently assessed by two reviewers, with disagreements resolved through discussion. The overall certainty of evidence was qualitatively evaluated considering risk of bias, consistency, directness, and precision of estimates.

### 2.5. Data Synthesis and Statistical Analysis

Data were pooled using random-effects models according to the DerSimonian and Laird method [30]. Prespecified subgroup analyses were conducted according to PH phenotype, study design (RCT vs. observational), and PADN technique. Meta-regression analyses were planned to explore sources of heterogeneity when sufficient data were available. Mean differences (MDs) with 95% confidence intervals (CIs) were calculated for continuous outcomes, and odds ratios (ORs) or risk ratios (RRs) for categorical outcomes [31].

When only medians and interquartile ranges were reported, means ± standard deviations were estimated using the method described by Wan et al. [32]. Heterogeneity was assessed using I^2^ statistics. Sensitivity analyses were performed by excluding individual studies. Publication bias was evaluated using funnel plots and Egger’s/Begg’s tests [33]. Analyses were conducted using Comprehensive Meta-Analysis and RevMan 8.1.1 [13,16]. Statistical significance was set at *p* < 0.05.

### 2.6. Protocol Registration

**Figure 1:**
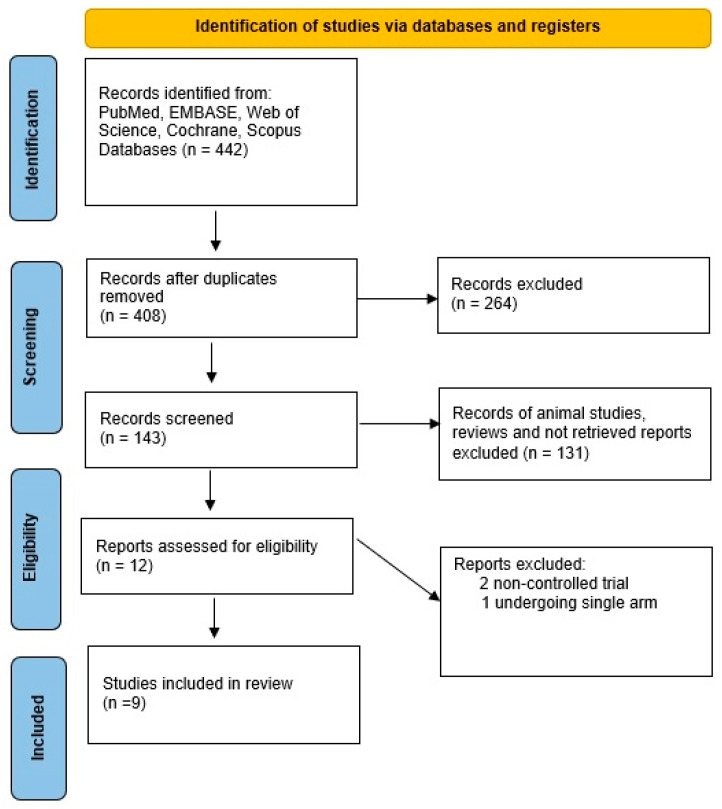
Preferred reporting items for systematic reviews and meta-analysis (PRISMA) flow diagram for the study selection process.

**Figure 2:**
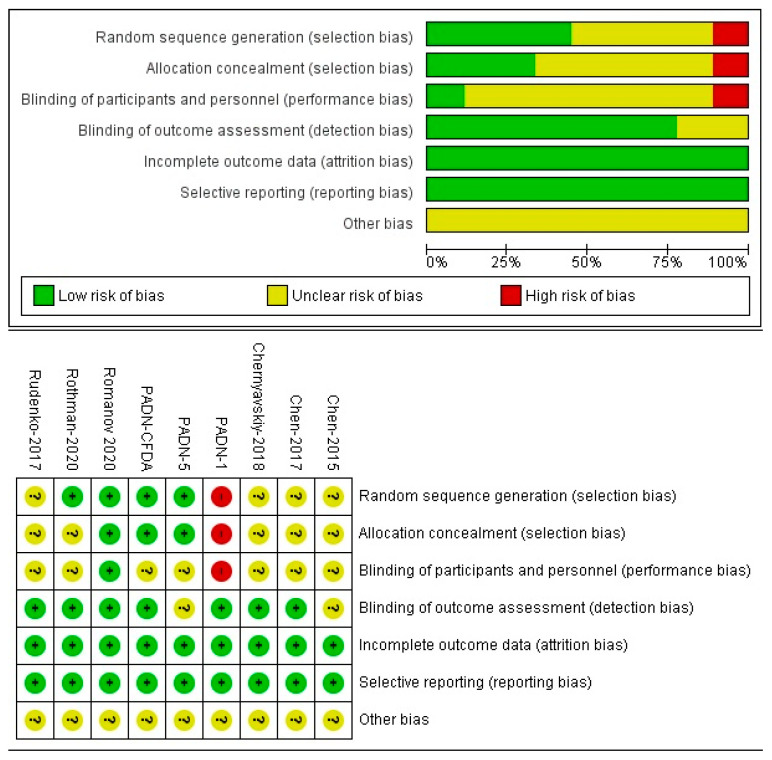
Literature quality evaluation, according to the risk of bias tool, the green represents “low risk”, the yellow represents “unclear risk”, the red represents “high risk”.

This systematic review and meta-analysis were conducted in accordance with the Preferred Reporting Items for Systematic Reviews and Meta-Analyses (PRISMA) 2020 guidelines [26]. Any disagreements between reviewers during study selection and data extraction were resolved through discussion. The review protocol was registered in PROSPERO (CRD420250652620) [27]. Reasons for full-text exclusion are detailed in the PRISMA flow diagram (Figure 1), and Figure 2 summarizes the risk-of-bias assessment of the included studies.

## 3. Results

### 3.1. Study Selection

The initial search of electronic databases and other sources identified 442 publications. After removal of 36 duplicates, 143 studies were screened based on titles and abstracts. Of these, 131 records were excluded, and the full texts of 12 articles were evaluated for eligibility. Following full-text review, nine studies were included in the meta-analysis: five single-arm prospective studies, one non-randomized clinical trial, and three randomized clinical trials, encompassing a total of 454 patients [13,14,15,16,17,18,19,20,21,22,23]. All clinical trials performed subgroup analyses comparing pre- and post-PADN outcomes within the same patient group, while studies with control groups [13,16,17,18,22] also reported between-group comparisons. Study characteristics are summarized in Table 1.

### 3.2. Patient Characteristics

Across the nine included studies, the mean patient age was 47.8 years (95% CI: 42.2–53.5 years); 57% were female (95% CI: 43–71%), and 79% were in NYHA functional class III or IV (95% CI: 61–90%) [13,14,15,16,17,18,19,20,21,22,23].

### 3.3. Functional Outcomes

#### 3.3.1. Six-Minute Walk Distance (6MWD)

**Figure 3:**
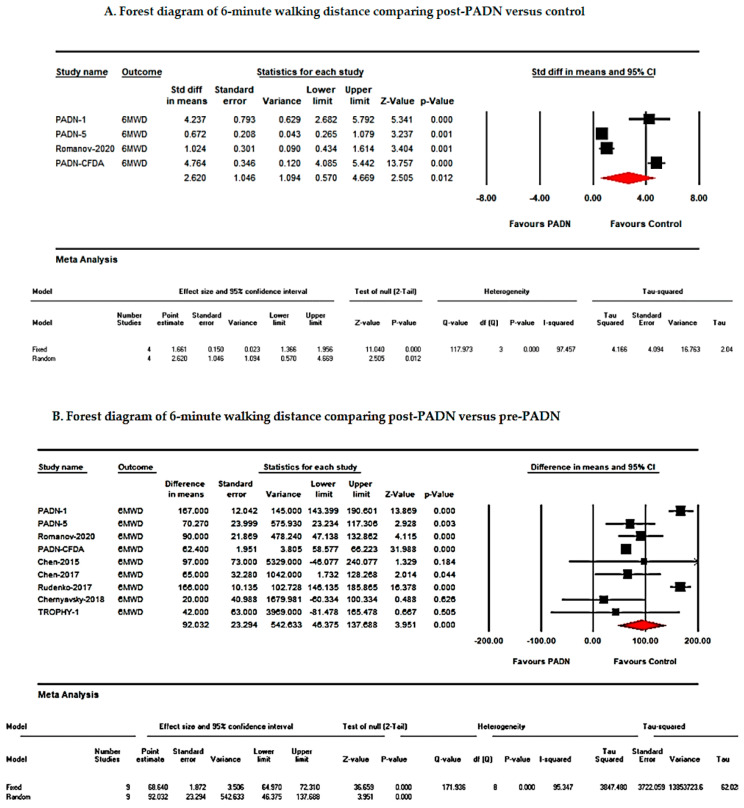
Forest plot of six-minute walk distance (6MWD) pre- and post-PADN in patients with pulmonary hypertension [14,15,21,23].

PADN therapy significantly improved 6MWD in patients with PH. Using a random-effects model, the pooled analysis demonstrated a mean difference of 92.03 m (95% CI: 46.37–137.68; *p* < 0.001; I^2^ = 95%) (Figure 3).

Standardized mean difference (Std diff in means) was 2.62 (95% CI: 0.57–4.66; *p* = 0.012; I^2^ = 97%). High heterogeneity was observed, likely attributable to differences in PH phenotypes, baseline disease severity, background medical therapy, and PADN procedural techniques.

#### 3.3.2. NT-proBNP

PADN was associated with a significant reduction in NT-proBNP levels (Std diff in means = −2.42; 95% CI: −4.71 to −0.12; *p* = 0.039; I^2^ = 98%).

### 3.4. Hemodynamic and Echocardiographic Outcomes

Pre- and post-PADN comparisons revealed significant improvements in pulmonary hemodynamics and right ventricular function:

- Mean Pulmonary Artery Pressure (mPAP): MD = −11.84 mmHg; 95% CI: −16.46 to −7.23; *p* < 0.001.
- Pulmonary Vascular Resistance (PVR): MD = −4.88 Wood units; 95% CI: −6.81 to −2.95; *p* < 0.001.
- Cardiac Output (CO): MD = 0.55 L/min; 95% CI: 0.30–0.81; *p* < 0.001.
- Right Ventricular Tei Index: MD = −0.15; 95% CI: −0.21 to −0.10; *p* < 0.001.
- Tricuspid Annular Plane Systolic Excursion (TAPSE): MD = 0.23 mm; 95% CI: −0.02 to 4.90; *p* = 0.07. Although a trend toward improvement in TAPSE was observed, the effect did not reach statistical significance, suggesting limited sensitivity of TAPSE as a marker of PADN-related RV functional changes.
- Right Ventricular Fractional Area Change (RV FAC): MD = 4.69%; 95% CI: 0.96–8.42; *p* = 0.014.

**Figure 4:**
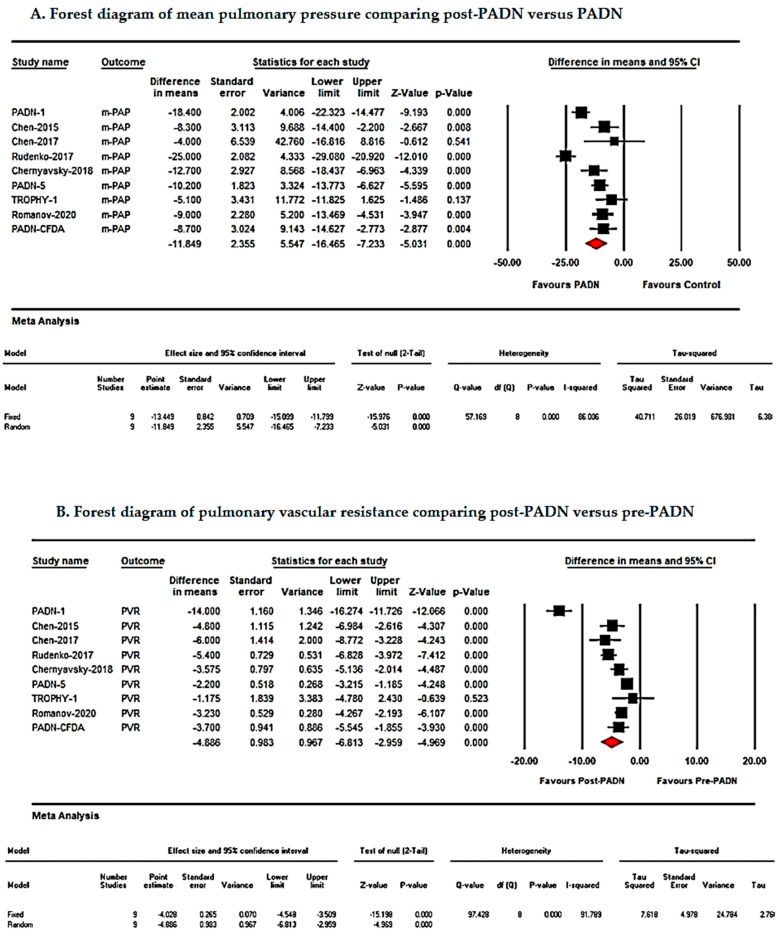

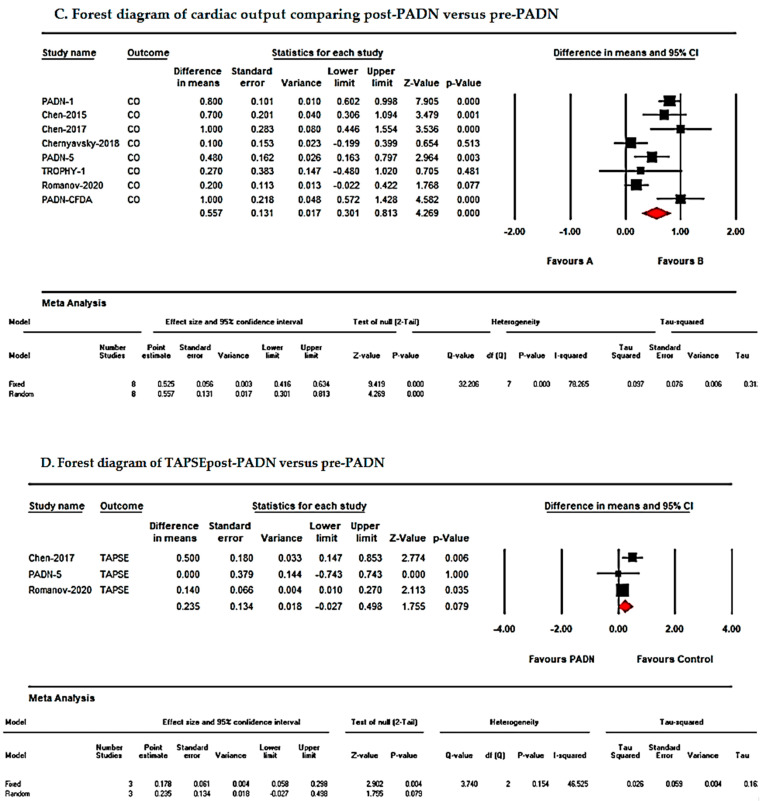
Forest plot of hemodynamic outcomes after PADN: (**A**) mean pulmonary artery pressure (mPAP); (**B**) pulmonary vascular resistance (PVR); (**C**) cardiac output (CO); and (**D**) TAPSE.

**Figure 5:**
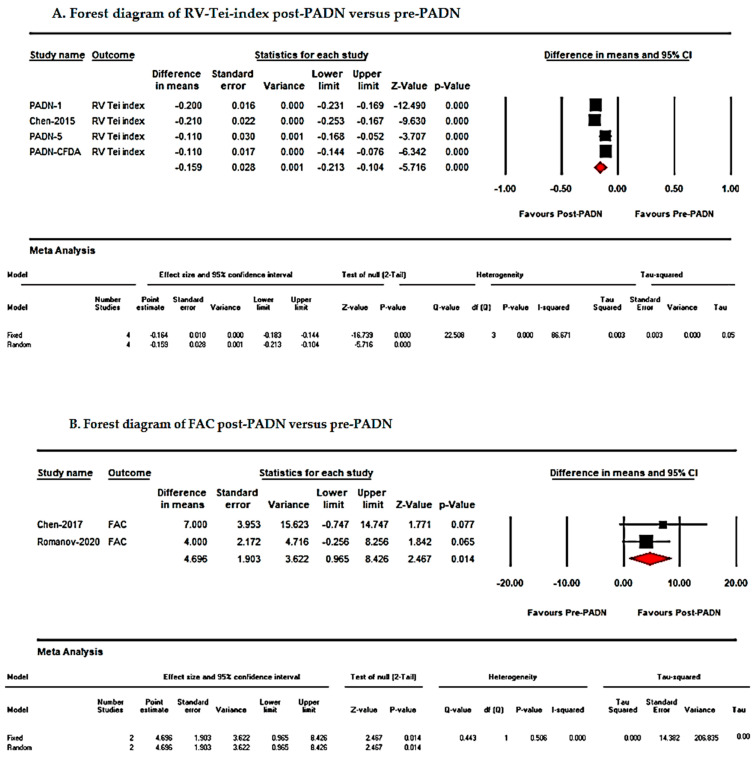

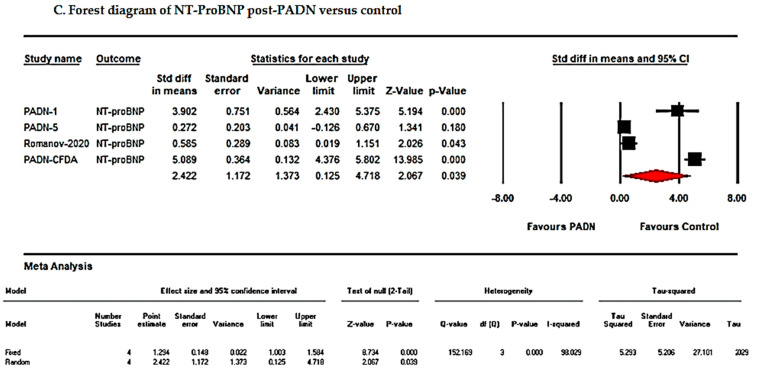
Forest plot of right ventricular functional and biomarker outcomes after PADN: (**A**) RV Tei-index, (**B**) RV fractional area change (FAC), and (**C**) NT-proBNP.

Due to high heterogeneity (I^2^ > 75%), a random-effects model was applied for all analyses. Forest plots illustrating these outcomes are presented in Figure 4 and Figure 5 and Table 2 [8,9,10,11,12].

### 3.5. Long-Term Clinical Outcomes

Clinical endpoints included PAH-related rehospitalization, clinical worsening, lung transplantation, and all-cause mortality. Across all studies, the following data was collected at the last follow-up:

- All-cause mortality: 26 patients (10%; 95% CI: 6–16%);
- Clinical worsening: 40 patients (16%; 95% CI: 8–30%);
- Rehospitalization: 34 patients (12%; 95% CI: 5–26%);
- Transplantation: 4 patients (2%; 95% CI: 1–5%).

Pooled analyses comparing PADN and control groups demonstrated:

- All-cause death: OR = 0.53; 95% CI: 0.24–1.18; *p* = 0.12; I^2^ = 0;
- Transplantation: OR = 0.20; 95% CI: 0.009–4.27; *p* = 0.30; I^2^ = 0;
- Clinical worsening: OR = 0.30; 95% CI: 0.15–0.60; *p* = 0.001; I^2^ = 0;
- Rehospitalization: OR = 0.07; 95% CI: 0.019–0.28; *p* < 0.001; I^2^ = 0.

**Figure 6:**
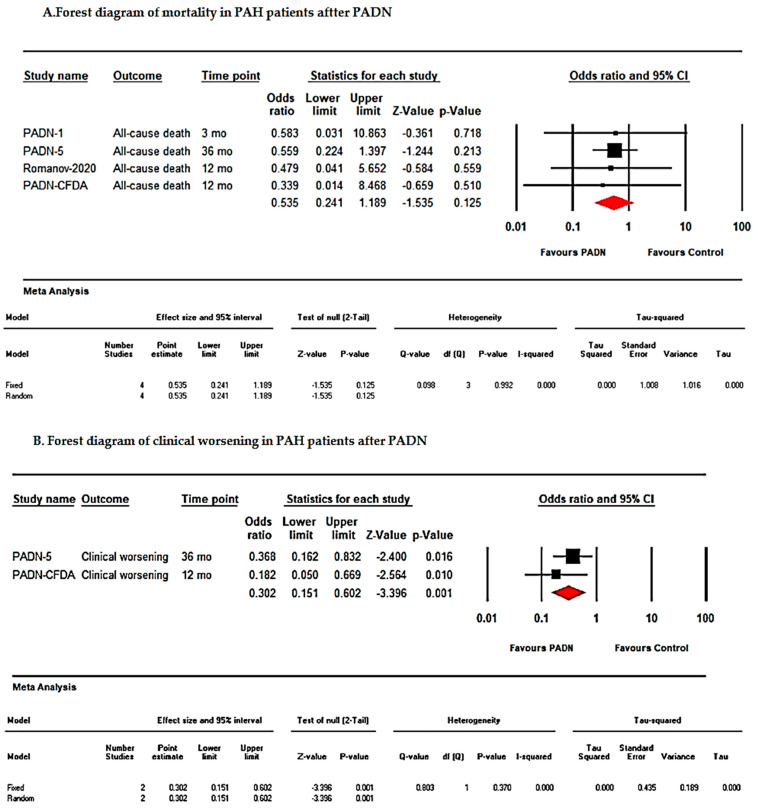

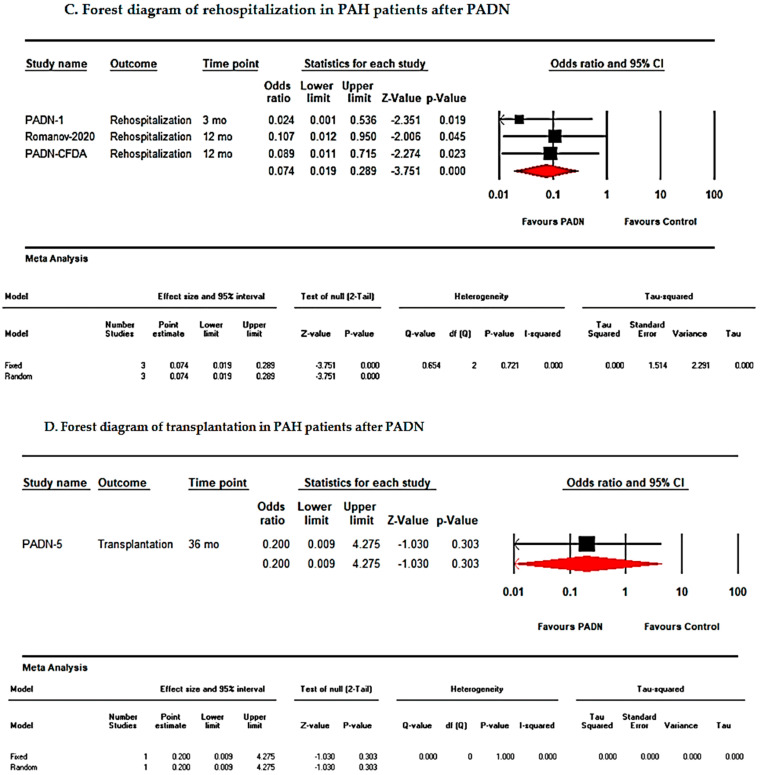
Forest plots of long-term clinical outcomes after pulmonary artery denervation (PADN). (**A**) All-cause mortality. (**B**) Clinical worsening. (**C**) Rehospitalization. (**D**) Lung transplantation. Pooled odds ratios (ORs) with 95% confidence intervals (CIs) are shown using fixed-effects models. Squares represent individual study estimates, and diamonds represent pooled effects.

These results indicate significant reductions in clinical worsening and rehospitalization in the PADN group, whereas differences in mortality and transplantation did not reach statistical significance (Figure 6).

## 4. Discussion

This systematic review and meta-analysis evaluated the efficacy of pulmonary artery denervation (PADN) in patients with pulmonary hypertension (PH) refractory to medical therapy. The pooled analysis demonstrated that PADN significantly improved right ventricular (RV) function and functional status, as evidenced by increases in six-minute walk distance (6MWD; Figure 3) and reductions in mean pulmonary artery pressure (mPAP; Figure 4 and Figure 5, Table 2). Additionally, PADN was associated with reductions in clinical worsening and rehospitalization (Figure 6), while effects on mortality and transplantation were minimal.

The central hypothesis underpinning PADN is that selective ablation of sympathetic nerves in the pulmonary artery interrupts pathological sympathetic signaling, reducing vasoconstriction, improving pulmonary hemodynamics, and alleviating RV overload. These effects may contribute to slowing disease progression in PH [8,9,11,12]. Consistent reductions in mPAP, pulmonary vascular resistance (PVR), and improvements in RV functional parameters observed in this analysis support this mechanistic rationale. Beyond hemodynamic unloading, PADN may exert disease-modifying effects through autonomic rebalancing and neurohumoral modulation. Experimental and translational evidence suggests that interruption of sympathetic afferent signaling reduces neurogenic vasoconstriction, attenuates inflammatory signaling cascades, and modulates the local renin–angiotensin–aldosterone system within the pulmonary vascular bed [8,9,10,11,12]. These mechanisms may facilitate reverse pulmonary vascular remodeling, improve endothelial function, and restore right ventricle–pulmonary artery coupling. Concomitant reductions in sympathetic tone may lessen right ventricular afterload sensitivity and improve ventricular–arterial coupling, thereby enhancing right ventricular efficiency beyond the effects of pulmonary artery pressure reduction alone.

The first-in-human PADN-1 trial by Chen et al. [13] demonstrated significant declines in pulmonary pressures and improved 6MWD among patients with pulmonary arterial hypertension. However, limitations included a highly selected patient population, high prevalence of long-term oxygen therapy, and predominance of male participants, which may have introduced survivor bias [10]. Subsequent trials, such as TROPHY-1 [19], enrolled patients on dual or triple oral therapy who did not respond to acute vasodilator testing. These studies showed improvements in 6MWD and PVR but did not evaluate optimal timing for PADN or long-term survival benefits.

Our pooled analysis corroborates these findings, showing reductions in mPAP, PVR, and NT-proBNP (Figure 5), likely reflecting decreased sympathetic activity and modulation of the renin–angiotensin–aldosterone system [8,9,10,11,12]. These hemodynamic improvements may enhance RV performance, cardiac output, and right ventricular fractional area change, contributing to improved exercise capacity and 6MWD [11,12]. Recent comparative evidence suggests that PADN may provide incremental functional and hemodynamic benefit when evaluated alongside contemporary therapeutic strategies, supporting its potential integration into evolving treatment algorithms [34].

The temporal profile of PADN-related benefits also warrants consideration. Available evidence suggests that early effects are predominantly hemodynamic, with reductions in mean pulmonary artery pressure and pulmonary vascular resistance observed shortly after the procedure. Improvements in functional capacity, including six-minute walk distance, and right ventricular performance tend to emerge over the intermediate term, likely reflecting progressive ventricular–arterial uncoupling reversal and improved RV adaptation. In contrast, longer-term outcomes such as reductions in clinical worsening and rehospitalization appear to become more evident with extended follow-up.

From a clinical standpoint, PADN does not necessitate a fundamentally distinct follow-up strategy compared with standard pulmonary hypertension management. Instead, post-procedural monitoring should remain aligned with guideline-based practice, including serial assessment of World Health Organization functional class, exercise capacity, and biomarkers such as NT-proBNP. Echocardiographic evaluation of right ventricular size and function remains essential, while repeat right heart catheterization may be considered in selected patients to reassess hemodynamic response. This integrated approach allows PADN to be incorporated into existing care pathways without requiring a separate surveillance paradigm.

The benefits of PADN were particularly evident when comparing pre- and post-treatment outcomes, as well as intervention versus control groups, with the latter demonstrating significantly lower rates of clinical worsening and rehospitalization (Figure 6). Nevertheless, high heterogeneity (I^2^ = 95%) highlights variability in patient populations, study designs, and follow-up durations, which may influence pooled estimates [24]. Updated pooled analyses have emphasized that variability in procedural technique and patient selection remains a major determinant of outcome heterogeneity, underscoring the need for standardization in future investigations [35].

Sham-controlled trials combining PADN with phosphodiesterase-5 inhibitors demonstrated that at one-year follow-up, PADN-treated patients experienced lower rates of clinical worsening and improved 6MWD compared with sham controls [20]. These results align with our meta-analysis, reinforcing the role of PADN in improving functional outcomes, while its impact on mortality and transplantation remains uncertain.

While pulmonary arterial hypertension remains the most studied phenotype, PH associated with left heart disease is the most prevalent form, representing 65–80% of cases [17,36,37]. Data on PADN in this population are limited, though PADN-5 demonstrated reductions in clinical worsening when compared with sildenafil and sham interventions [17]. Similarly, small studies in patients with residual chronic thromboembolic PH after pulmonary endarterectomy have shown improvements in hemodynamics and functional capacity [22,23]. These findings suggest that PADN may have applicability across diverse pulmonary hypertension phenotypes; however, larger trials are required to confirm these observations. Ongoing prospective investigations are expected to further clarify feasibility, safety, and patient selection strategies in medication-refractory disease.

This analysis represents the most updated synthesis of PADN outcomes, incorporating both randomized and prospective observational studies. PADN emerged as a strong independent predictor of freedom from clinical worsening, supporting its potential as a therapeutic option for selected patients with medication-refractory PH [11]. Challenges remain regarding optimal patient selection, procedural technique, and long-term durability of effects. Furthermore, the majority of included studies were single-arm interventional trials, limiting the certainty of evidence and introducing potential selection bias.

Several limitations should be considered when interpreting the results of this meta-analysis. First, the majority of included studies were single-arm prospective trials, with only a limited number of randomized controlled trials available, thereby increasing susceptibility to selection bias and limiting causal inference. Second, substantial heterogeneity was observed across studies in terms of patient populations, pulmonary hypertension phenotypes, disease severity, and background medical therapy, which may have influenced pooled effect estimates. Third, follow-up durations varied considerably, and long-term outcomes beyond one year remain largely unknown, precluding definitive conclusions regarding survival benefit and disease modification. Fourth, procedural heterogeneity, including differences in PADN techniques, energy delivery methods, anatomical targeting, and operator experience, may have affected treatment consistency and outcome reproducibility. Fifth, restriction to English language publications introduces potential language bias. Sixth, publication bias cannot be fully excluded despite funnel plot assessment, particularly given the predominance of single-center interventional studies. Finally, as with all meta-analyses, the reliability of conclusions is inherently dependent on the quality and design of the included primary studies. These limitations underscore the need for large-scale, multicenter, sham-controlled randomized trials with standardized PADN protocols and long-term follow-up.

Pulmonary artery denervation may serve as a complementary interventional option in carefully selected patients with pulmonary hypertension who remain symptomatic despite optimized guideline-directed medical therapy. In this context, a more refined clinical characterization of potential responders is warranted. Based on the patterns observed across the included studies, patients in World Health Organization functional class III–IV with persistently elevated pulmonary vascular resistance and mean pulmonary artery pressure appear to represent a subgroup with a higher likelihood of benefit. The presence of ongoing right ventricular (RV) dysfunction, impaired RV–pulmonary arterial coupling, and recurrent clinical worsening or hospitalization may further identify patients in whom autonomic modulation through PADN could provide incremental therapeutic value.

Conversely, in patients with advanced and potentially irreversible RV failure, severe comorbidity burden, or end-stage pulmonary vascular disease, the expected benefit of PADN may be attenuated. Therefore, PADN should be considered as a targeted adjunctive therapy rather than a universal intervention. Although PADN remains investigational, its integration into multidisciplinary pulmonary hypertension management strategies may offer a novel approach to addressing unmet therapeutic needs in refractory disease phenotypes. Future research should focus on large-scale randomized studies to better define responder phenotypes, identify potential “super-responder” profiles, and determine long-term efficacy and durability. Emerging reports describing combined interventional strategies suggest that integrating PADN with structural or valvular therapies may offer a comprehensive approach to managing pulmonary vascular load and right-sided cardiac dysfunction in complex disease phenotypes.

## 5. Conclusions

In patients with pulmonary hypertension refractory to medical therapy, pulmonary artery denervation is associated with improvements in functional capacity, pulmonary hemodynamics, and right ventricular performance. PADN also appears to reduce clinical worsening and rehospitalization rates; however, its impact on long-term survival and transplantation remains uncertain due to limited and heterogeneous evidence. Although PADN represents a promising adjunctive therapeutic strategy, the current body of evidence is derived largely from small-scale and heterogeneous studies, with a limited number of randomized controlled trials. Therefore, PADN should be considered investigational, and its routine use in clinical practice cannot yet be recommended. Future large-scale, multicenter, sham-controlled randomized trials are required to better define patient selection, standardize procedural techniques, and establish long-term efficacy and safety.

## Figures and Tables

**Table 1:** Characteristics of included studies evaluating PADN in pulmonary hypertension (Columns: Author, Year, Study Design, Sample Size, PH Type, Follow-up Duration, and PADN Technique).

Author	Study Type	WHO Group Class	Intervention Group	Control Group	Population	Follow-Up	Outcomes
Chen et al., 2013 (PADN-1) [13]	Non-randomized clinical trial	Group I	Percutaneous PADN (RFA). Before the procedure, patients received diuretics and beraprost, either sildenafil, bosentan, or digoxin. They were discontinued after PADN.	Before enrollment, patients received diuretics and beraprost, either sildenafil or bosentan or digoxin.	21	3 mo	mPAP,6MWD, RV Tei index and adverse effects
Chen et al., 2015 (PADN-Phase-II) [14]	Single-arm-open-label prospective study	Group I Group II Group IV	Percutaneous PADN (RFA).	N/A	66	6 mo, 12 mo	mPAP,6MWD, CO, PVR and RV Tei index
Chen et al., 2017 [15]	Single-arm prospective study	Group I	Percutaneous PADN (RFA).	N/A	40	1 wk, 3 mo, 6 mo, 12 mo	mPAP,6MWD and PVR
Rudenko et al., 2017 [23]	Single-arm prospective study	Group IV	PADN via simplicity denervation system.	N/A	12	3 mo	mPAP,6MWD and PVR
Chernyavskiy et al., 2018 [21]	Single-arm prospective study	Group IV	Percutaneous PADN (RFA).	N/A	16	days	mPAP,6MWD, CO and PVR
Zhang et al., 2019 (PADN-5) [17]	Randomized, sham-controlled trial	Group II	Percutaneous PADN (RFA). Standard anti-heart failure medications were administered. In the three months prior to the hospitalization, patients had stopped using any drugs that targeted PAH.	Sildenafil + Sham PADN. Standard anti-heart failure medications were administered. Patients were off any medications targeting PAH in the 3 months before admission.	98	6, 12, 36 mo	mPAP,6MWD, CO, PVR and adverse events
Rothman et al., 2020 (TROPHY-1) [19]	Single-arm, non-randomized, non-controlled, multi-center, prospective study	Group I	Percutaneous PADN (HEU) (TIVUS System). Patients who did not respond to acute vasodilator testing received dual oral or triple non-parenteral therapies.	N/A	23	4, 6 mo	mPAP,6MWD, CO, PVR, procedure-related adverse events, disease progression, and mortality
Romanov et al., 2020 [22]	Randomized clinical trial	Group IV	Percutaneous PADN (RFA)	Riociguat + Sham PADN.	50	12 mo	mPAP,6MWD, CO and PVR
Zhang et al., 2022 (PADN-CFDA) [16]	Randomized, sham-controlled, multi-center clinical trial	Group I	Percutaneous PADN (RFA) + PDE5 inhibitor. Prior to the trial, patients were clinically stable for at least 30 days without receiving medical PAH therapy.	Sham PADN + PDE5 inhibitor. Prior to the trial, patients were clinically stable for at least 30 days without receiving medical PAH therapy.	128	6 mo, 12 mo	mPAP,6MWD, CO, PVR and clinical worsening

**Table 2:** Pooled hemodynamic and right ventricular functional outcomes following PADN (*Columns: Outcome, MD, 95% CI, p-value, I*^2^).

				Follow-Up:			
		Number of Studies Included		Mean Difference or Relative Risk (95% CI)	*p* Value	I^2^ (%)	*p* for Heterogeneity
Functional Status							
6MWD (m)	Mean	9	Mean difference	92.03 (46.3 to 137.6)	<0.001	95	<0.001
Echocardiographic data							
mPAP	Mean	9	Mean difference	−11.84 (−16.46 to −7.23)	<0.001	86	<0.001
TAPSE (mm)	Mean	3	Mean difference	0.23 (−0.02 to 4.90)	0.07	46	0.15
PVR	Mean	9	Mean difference	−4.88 (−6.81 to −2.95)	<0.001	91	<0.0001
RV Tei-index	Mean	4	Mean difference	−0.15 (−0.21 to −0.10)	<0.001	86	<0.0001
RV FAC (%)	Mean	2	Mean difference	4.69 (0.96 to 8.42)	0.014	0	0.50
CO (L/min)	Mean	8	Mean difference	0.55 (0.30 to 0.81)	<0.001	78	<0.0001

6MWD = 6MWD,6 min walk distance; CO, cardiac output; mPAP, mean pulmonary artery pressure; PADN, pulmonary artery denervation; PVR, pulmonary vascular resistance; RV FAC, right ventricle fractional area change.

## Data Availability

All data generated or analyzed during this study are included in this article. Further inquiries can be directed at the corresponding authors.

## Supplementary Materials

The following supporting information can be downloaded at https://www.mdpi.com/article/10.3390/jcm15072619/s1. Table S1: PRISMA Checklist.
